# Eutectogels as Delivery Media for Therapeutic Metal Complexes: What Are the Benefits?

**DOI:** 10.3390/gels12010065

**Published:** 2026-01-11

**Authors:** Varvara O. Veselova, Svetlana V. Revtovich, Vitalia V. Kulikova, Arina D. Filippova, Kseniya A. Koshenskova, Nikolay N. Efimov, Irina A. Lutsenko, Marina A. Uvarova

**Affiliations:** 1 Kurnakov Institute of General and Inorganic Chemistry, Russian Academy of Sciences, 119991 Moscow, Russia; 2 Engelhardt Institute of Molecular Biology, Russian Academy of Sciences, 119991 Moscow, Russia

**Keywords:** eutectogel, silica, antibacterial activity, transdermal penetration, deep eutectic solvent, reline

## Abstract

Drugs and drug candidate compounds commonly suffer from poor solubility and permeability. One promising strategy to mediate these drawbacks is use of novel solvents, such as deep eutectic compositions. The present research aims to determine the applicability of this approach for therapeutic metal complexes on the example of [Cu(Fur)_2_(Phen)] (Fur = furoate-anion, Phen = 1,10-phenantroline) and [Cu(Fur)_2_(Neoc)(H_2_O)] (Fur = furoate-anion, Neoc = 2,9-dimetyl-1,10-phenanthroline) with molar weight of appx. 500 Da. Interaction of the metal complexes with the deep eutectic solvent (DES) reline was studied using electron paramagnetic resonance (EPR). Minimal inhibitory concentrations of the complexes dissolved in DES and dimethyl sulfoxide (DMSO) were determined and found to be equivalent in both solvents. That is, use of reline as a solvent did not alter the functional properties of the metal complexes. Changes in the transdermal permeation of the complexes in DMSO and DES were assessed using a Franz diffusion cell. It was discovered that depending on the structure of the complex, the permeability might either increase (from 15 to 30%) or decrease (from 13 to 8%) with changes in the solvent, and this can be used to develop dosing strategies. Therapeutic eutectogels were successfully produced by impregnating SiO_2_ nanoparticles with the metal complex solution in DES, facilitating convenient topical application.

## 1. Introduction

**Figure 1:**
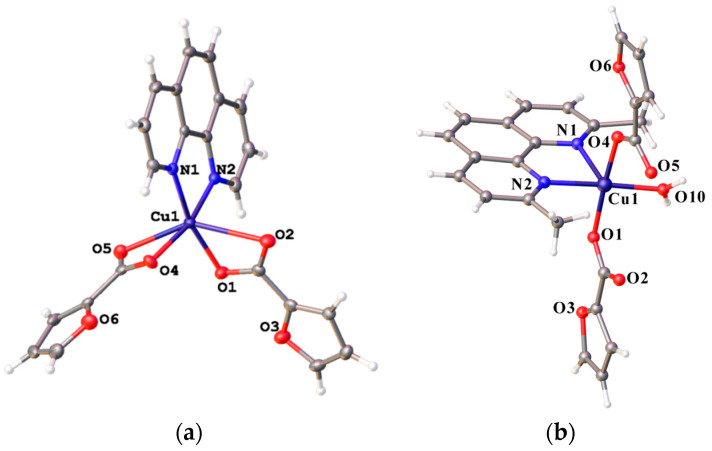
Structure of the antibacterial Complex A (**a**) and B (**b**).

The Biopharmaceutics Classification System (BCS) [1] categorizes drugs on the basis of their solubility and permeability. The success of an active pharmaceutical ingredient (API) in pharmaceutical development is largely defined by these two parameters [2,3,4]. Poor solubility of drug candidate compounds in aqueous media is a significant bottleneck in drug development [5]. Poor solubility hampers systemic absorption [6,7] and complicates formulation processes, dosing strategies, and storage stability [8,9]. In 2023, the share of insufficiently soluble drugs in aqueous media was estimated to be 60–70% [10]. One of the types of drugs with poor aqueous solubility are the metal complexes. Platinum complexes, many of which suffer from insufficient water solubility, are extensively used in cancer therapy [11]. Plenty of poorly soluble metal complexes are considered as drug candidates for therapy of anemia [12], cancer [13], tuberculosis [14], etc. For example, copper(II) coordination compounds with oligopyridine ligands show promising antimicrobial properties [15,16], and copper(II) furancarboxylate complexes show promising anti-tuberculosis and antibacterial activity [17,18]. Two complexes were chosen as the model objects in the present study: [Cu(Fur)_2_(Phen)] (Complex A) [17] and [Cu(Fur)_2_(Neoc)(H_2_O)] (Complex B) [18]. The respective structures of these complexes are shown in Figure 1. Both of these complexes are poorly soluble in water and are reported to have pronounced antibacterial activity and potential application as tuberculosis therapy [18].

Various strategies for solubility enhancement have been suggested over the years [19], such as salt formation, co-crystallization, amorphization, nanoformulations, solid dispersions, use of solubilizing agents [20,21,22,23], etc.

One of the emerging strategies that has been recognized as promising is the development of novel solvent systems based on ionic liquids (ILs). However, first generation of IL based on ammonium, imidazolium, phosphonium, pyridinium, and pyrrolidinium cations exhibit acute toxicity and cause skin irritation [24,25] which limits their applications in drug delivery systems. Deep eutectic solvents comprising biocompatible components have allowed us to overcome these limitations and formulate novel solvents with excellent biocompatibility profiles and adequate biodegradation features. Not only do the DESs help to improve solubility of the API, but they also reportedly help to increase bioavailability and perform as penetration enhancers by overcoming tissue barriers [26]. DESs are generally cheaper and easier to prepare compared to first-generation ILs, too. A detailed discussion of ILs and DES differences and similarities as well as formation mechanisms of these solvents can be found elsewhere [27].

Solubility enhancement in DES is associated with their ability to form strong intermolecular interactions, including hydrogen bonding, van der Waals forces, and electrostatic interactions [28]. However, the process of API solvation in DESs is not yet fully understood and remains highly system specific [29]. To date, the researchers’ attention was mainly focused on common APIs such as analgesics (paracetamol [30], ibuprofen [31]), nonsteroidal anti-inflammatory drugs (celecoxib [32], indomethacin [33], etc.), and other small molecules [34,35]. To the best of our knowledge, to date there is little to no data on the speciation of therapeutic metal complexes in DESs, though it has been reported that the DESs components (e.g., urea or chloride anion) might take place in the coordination shell of the metal, as reported in [36,37]. So, the present work aims to establish whether it is possible to use DES to solubilize metal complexes or if decomposition of the original complex will be observed due to interaction with the DES components. For the present research, reline was chosen as DES. This DES comprises choline chloride and urea in a molar ratio of 1:2 and is one of the best-studied DES compositions [38,39]. Reline meets the following requirements: (1) accessibility of components and ease of synthesis; (2) well described properties; (3) pharmacological inactivity; (4) no toxicity; (5) biodegradability; (6) unlimited solubility in water; and (7) has a pH that is relatively close to physiological values [40]. Unlike one of the most popular DESs used for drug delivery, choline geranate (CAGE) [40], reline does not possess intrinsic antimicrobial activity, which would make it possible to evaluate the performance of the target metal complex dissolved in the DES on its own [41]. Additionally, the pH of reline does not change significantly upon the addition of water up to 50 wt.% [42], which makes it more suitable for application on skin.

The goal of the present work is to determine the type of interaction that exists between the metal complex and DES, the effect of this interaction on the therapeutic properties of the metal complex, and whether the DES increases the permeability of the therapeutic complex. The second task is to develop a technique for obtaining a gel containing DES and an antibacterial metal complex to facilitate application to the skin.

## 2. Results and Discussion

The chosen complexes do not undergo visually observable hydrolysis or decomposition in reline. Solubility of the complexes in reline surpasses 5 mg/g, which is more than enough for therapeutic applications.

However, it is known that Cu(II) ion can be coordinated with components of ionic liquid [43,44] and complexes of copper might undergo ionic exchange with components of ionic liquids [45]. One of the simpler approaches to studying the complexation processes is UV-vis spectroscopy. For example, 1,10-Phenanthroline has typical absorption lines at 220 and 265 nm. The shift of the absorption line from 265 nm to 275 nm is evidence of coordination with copper [46,47]. In this particular case, this method was not applicable, because reline absorbs in the UV-vis region [48] and it was not possible to collect an informative spectrum.

In previous work, it has been shown that interaction of [CuCl_4_]^2−^ ion with choline chloride lends the solution a bright yellow color [45]. The fact that the color of the complexes’ solutions in DES and DMSO are the same in the present case acts as indirect evidence that the coordination of Cu(II) ion remains unchanged. To additionally assess changes in the copper ion coordination, we have employed EPR spectroscopy.

**Figure 2:**
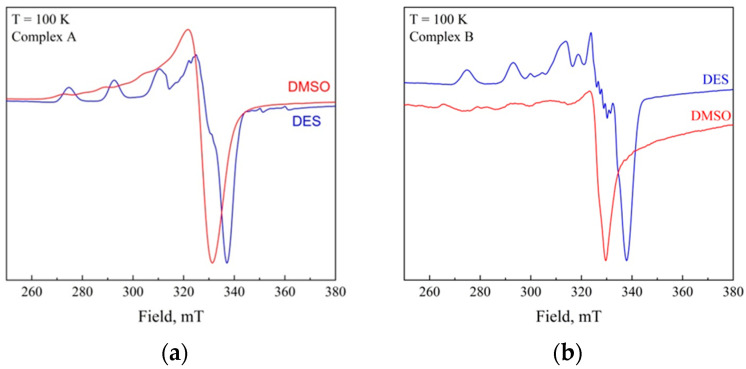
EPR powder spectra of the antibacterial complexes A (**a**) and B (**b**) in DMSO and reline at 100 K.

The EPR spectra (Figure 2) of both complexes in both of the studied solvents confirm that the Cu(II) center is axial. These spectra display a typical copper ion signal with four lines in the parallel region arising from the hyperfine coupling of the electron spin of Cu(II) with its nuclear spin. The obtained spectra are characteristic of an isolated Cu^2+^ ion with axial symmetry in the form of an octahedron with various tetragonal distortions and localization of the unpaired electron on the $3d_{x^{2}-y^{2}}$ orbital in the equatorial plane [49].

In the case of reline complex solutions, superhyperfine splitting is observed. This is most likely to be caused by an interaction with ^14^N atoms present in both urea and choline. Ligation of copper complexes with nitrogen-coordinating solvents with high donicity numbers such as pyridine [50,51,52], N,N-dimethylformamide (DMFA), N,N-dimethylacetamide (DMAA) [53], and formation of adducts has been reported in the literature. The donicity number value of reline (DN = 43.4) [54] exceeds that of pyridine (DN = 33.1) and DMFA (DN = 30.9) [55], so interaction between reline and copper ion is quite probable. However, the geometry of complexes remains unchanged and the elongated tetragonal bipyramid (axially distorted octahedral) coordination is preserved.

EPR spectra provide evidence of solvent–solute interactions between the therapeutic complexes and reline. It is important to note that the EPR spectra were acquired from frozen samples at 100 K to achieve better resolution. So, the observed interactions might be less pronounced in liquid solutions. To assess whether these interactions affect functional properties of the complexes, minimal inhibitory concentration (MIC) of complexes in reline, water, and DMSO was determined. Both tested compounds exhibit consistent antibacterial activity against *S. aureus* regardless of solvent type and the MIC value does not depend on the chosen solvent. That is, reline and the complex–reline interaction did not affect the antibacterial properties of the compounds. Complex B inhibits bacterial growth at a micromolar concentration of 6.3 µM, which is comparable to gentamicin (Table 1). Higher deviation (error value) obtained for the samples dissolved in reline might be caused by higher viscosity of the solution.

To assess the contribution of reline to antibacterial activity, its effect on *S. aureus* survival was additionally tested. The water stock solutions of tested complexes were prepared in a range of concentrations, as indicated in the Experimental section, at a constant DES concentration (1% of total well volume). The obtained MIC values of such solutions were identical to the MIC values determined in pure DMSO and reline, and the 1% DES solution in water was neither toxic to bacteria nor promoted bacterial growth (Table 1). Thus, any observed antibacterial action can be attributed solely to the activity of the complexes.

A solvent which is used to improve the poor solubility of the drug can at the same time act as a chemical permeation enhancer and a transdermal drug delivery system that can improve the biopharmaceutical performance of the drug across the skin barrier with minimal toxicity and/or irritation. As discussed in the Introduction, permeability of the therapeutic substance through the skin barrier is a key parameter along with solubility. The molecular size of the drug is a major factor that influences permeation because molecules with a weight greater than 500 Da cannot cross the skin by passive diffusion. The chosen model Complexes A and B have molecular weights of 466 and 494 Da, respectively, which is close to the approximate “borderline” value of 500 Da. DESs are known to improve permeation through the skin barrier, so a comparison of the complexes permeation in different solvents was carried out using a Franz cell.

**Figure 3:**
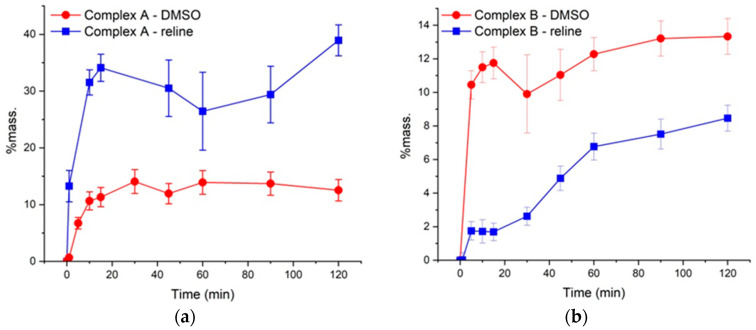
Concentration of Cu(II) relative to the initial loading of Complex A (**a**) and Complex B (**b**) in DMSO and reline vs. time in the receiver phase after passing through a Logan’s Permeation-Barrier Membrane at 37.5 °C. The error bars represent the standard deviation of the concentration.

Figure 3a,b shows the wt.% of copper ions released from the initial loading of the complex solution. The complex was released from 400 μL of either DMSO or reline into an acceptor chamber with a volume of 10 mL through a membrane imitating skin and the concentration of copper ions was determined by ICP-OES. The kinetic curves of release through the membrane simulating skin reached a plateau over the studied period of time (2 h).

Surprisingly, despite very similar structures of the tested therapeutic complexes, reline had opposite effect on their permeation rate. Use of Franz cell yielded results with a large error margin, so values of permeation constants could not be determined, but qualitative assessment of permeation remains possible. In case of Complex A, use of reline led to a twofold increase in copper content in the acceptor chamber. The copper concentration reached a plateau at ~15% when DMSO was used as a solvent, and at ~30% in the case of reline solution. A plateau was reached within the initial 20 min of observation, and a high initial release rate can be noted for both solutions.

In the case of complex B, the permeation was overall worse, reaching ~13% in case of DMSO solution. Use of reline caused a decrease in the permeation to ~8%. However, in this case, reline affected the initial permeation rate and the concentration of copper was increasing more steadily. The plateau was achieved in 15 min in the case of DMSO and in 60 min in the case of reline. The observed effect can be used to adjust the dosing to the desired rate.

After ensuring stability and preserved antibacterial activity of the complexes in reline and sufficient penetration of the active component through the skin barrier, the question of the dosage form was addressed. The therapeutic formulation needs to be applied to the target area and sufficiently prolonged treatment duration needs to be ensured. Liquid formulations are not the most convenient for this purpose. Creation of ionogels for medical applications, such as drug delivery and wound healing, has become a more actively researched topic in recent years [56,57]. To date, there are only isolated reports in the literature about the creation of eutectogels and very little research was performed on the possible functional uses of such DES-based materials.

To the best of our knowledge, no synthetic method for production of SiO_2_-based eutectogels has been reported to date. In general, two main approaches to production of ionic gels can be summarized from literature: gelation “in-situ” using the ionic liquid as the solvent; and impregnation/infusion of silica nanoparticles with the chosen IL [58].

Reportedly, gelation “in situ” yields more consistent and reproducible results in the case of first-generation ILs, so an attempt was made to adapt this approach, in order to produce eutectogels. However, it was quickly discovered that reline is immiscible with the most common silica precursors: TMOS, TEOS, and MTMS. Stirring allowed the production of stable emulsions in some of the tested systems. Gels were successfully obtained in systems TEOS-reline-CH_3_COOH, TMOS-reline-HCl, and TMOS-reline-CH_3_COOH (Table 2).

In the pilot experiments (Table 2), 1 mL of silica precursor was mixed with 1 mL of reline and 1 mL of gelation catalyst was added. The three systems listed above in which gel formation was observed were additionally tested with varied quantities of the gelation catalyst. Addition of 0.25 mL of the catalyst proved to be insufficient in all cases and the obtained gels were soft and not monolithic. Increasing the quantity of catalyst to 0.5 mL led to a significant increase in gelation time from 40 h to approximately one month. So, 1 mL of catalyst was chosen as the optimal amount.

In order to produce complex-containing eutectogels, solutions of Complexes A and B in reline with concentration of 5 mg/g were prepared and those solutions were used for the synthesis. However, addition of the catalyst led to visible precipitation and a change in color from blue to green in the case of Complex A and from brown-ish to green in the case of Complex B. Thus, the suggested synthetic route is not fit for production of therapeutic eutectogels containing copper complexes. However, it is still applicable for synthesis of silica-based eutectogels which might be of interest for production of extraction materials, inert synthetic templates, and other materials in the future.

**Figure 4:**
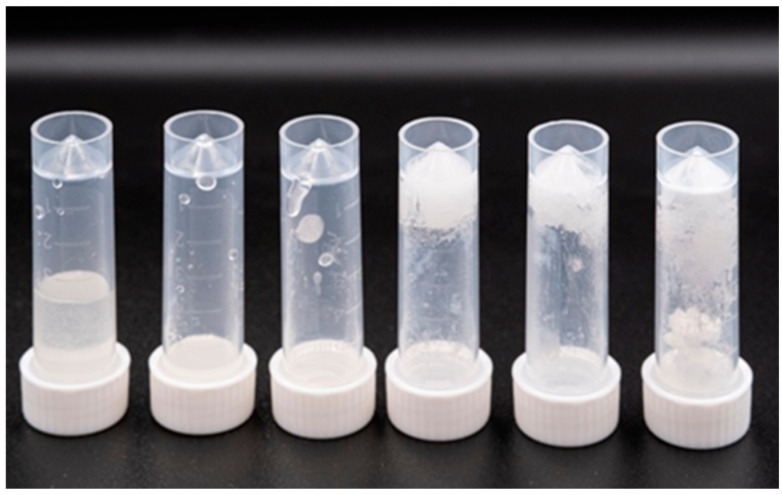
Dispersions of hydrophobic A380 silica nanoparticles in reline with particle concentrations increasing from left to right: 1 wt (%); 3 wt (%); 5 wt (%); 7 wt (%); 10 wt (%); and 12 wt (%).

To avoid the introduction of superfluous components, an impregnation route for eutectogel production was tested. Previously for the example of imidazolium-based ionic liquids, it was shown that such hydrophilic silica nanoparticles are preferable for formation of ionogels via the impregnation route [59]. In the present work Aerosil A380, a commercially available hydrophilic pyrogenic silicon oxide with a specific surface area of 380 m^2^/g, was chosen for preparation of eutectogel. Addition of only 7 wt.% of silica nanoparticles to reline, led to gel formation, as can be seen in Figure 4. Increasing the wt (%) of A380 can be used to adjust viscosity of the gel. Complexes A and B can be introduced into the gel in a wide variety of concentrations without compromising integrity of the complexes.

Reline, as well as SiO_2_ nanoparticles used for creation of the gel, are intentionally chosen to be inert. They are not intrinsically antibacterial or otherwise pharmaceutically active, which allowed us to easily evaluate the properties of the complexes in these media. In future research, a system comprising three independently therapeutic components—the drug, the solvent, and the gel matrix—can be designed and synergism of the components might be studied. Additionally, release rate of the therapeutic component from the eutectogel with different loading of SiO_2_ nanoparticles can be studied. The loading of the nanoparticles can be used as another parameter which can help adjust the release rate to the desired value and thus customize the dosing.

## 3. Conclusions

The main objective of the present work was to determine whether DES can be used for enhancement of solubility and permeability of therapeutic metal complexes. By using EPR study of copper complexes solutions in reline, interaction of the DES with the copper ion was confirmed. But this interaction did not affect the functional properties of the complexes. That is, the minimal inhibitory concentration of both complexes in DES and DMSO was determined and remained equal.

Study of the permeation rate of the complexes through a membrane imitating skin yielded unexpected results and demonstrated that using DES does not universally lead to increased permeation of the therapeutic complex. Based on this data, a careful choice of solvent might be used to adjust the pharmacokinetics to the desired rate. The obtained results confirm that the use of DES for delivery of therapeutic metal complexes is a viable strategy.

Two approaches to production of eutectogels loaded with the therapeutic complexes have been tested. A methodology for gelation of TMOS, TEOS, and MTMS precursors “in situ” using reline as a solvent has been successfully developed and SiO_2_-based eutectogels have been produced by this route for the first time. However, this synthetic route could not have been used for incorporation of copper complexes, because addition of gelation catalyst caused their decomposition. Thus, a eutectic gel containing antibacterial copper complexes, which can potentially be used as a transdermal antimicrobial material, has been produced via the impregnation route by adding 7 wt.% of hydrophilic silica nanoparticles.

## 4. Materials and Methods

### 4.1. Synthesis

Deep euthectic solvent reline was prepared by mixing 5.6 g of Choline Cloride (CAS-No.: 67-48-1, Sigma Aldrich, St. Louis, MO, USA, ≥98%) and 4.8 g of Urea (CAS-No.: 57-13-6, Sigma Aldrich, ≥99.5%) and keeping the mixture at 80 °C for 6 h or until a transparent viscous liquid is formed.

The complexes [Cu(Fur)_2_(Phen)] (Complex A) [17] and [Cu(Fur)_2_(Neoc)(H_2_O)] (Complex B) [18] were synthesized according to the procedures described in the cited sources. Solutions of the complexes in reline were prepared under ultrasound while heating the solution to 80 °C to reduce its viscosity.

For synthesis of eutectogels by gelation “in-situ,” reactants were used as follows: tetraethyl orthosilicate (TEOS, Sigma Aldrich, reagent grade, 98%), tetramethoxysilane (TMOS, 98%, Merck, Rahway, NJ, USA) and methyltrimethoxysilane (MTMS, 98%, Macklin, Shanghai, China), 0.01 M solution of hydrochloric acid (Sigma Tec, extra-pure grade, 36.5%), and acetic acid (Himmed, 99.9%, Moscow, Russia). The synthetic procedure was adapted from [60]. In a typical procedure, 1 mL of reline or complex solution in reline was added to 1 mL of TMOS, MTMS, or TEOS while stirring, and finally 0.25, 0.5, or 1 mL of acid solution was added. The obtained mixtures were observed for 48 h and in the case of gel formation, the gels were aged and observed for a month.

For preparation of eutectogels by the impregnation method, the solution of Complexes A and B in reline was loaded onto hydrophilic A380 silica powder at a chosen mass percentage.

### 4.2. Cell Culture

*Staphylococcus aureus* ATCC 29213 strain chosen for antibacterial testing was sourced from the American Type Culture Collection (ATCC; Rockville, MD, USA). The stock suspension of *S. aureus* was 1 × 10^8^ cells per mL.

### 4.3. Antibacterial Activity Evaluation

The MIC was determined in accordance with the Clinical and Laboratory Standards Institute (CLSI)-recommended methods M7-A9 [61] and M100-A30 [62]. Three independent experiments with four replicates each were performed to ensure reproducibility. Stock solutions of the complexes [Cu(Fur)_2_(Phen)] (Complex A) [17] and [Cu(Fur)_2_(Neoc)(H_2_O)] (Complex B) [18] were prepared in distilled water, DMSO, and reline. The concentrations of the tested substances in stock solutions were 5 mg/mL for Complex A and 2.5 mg/mL for Complex B. The tested concentration for Complex A ranged from 0.1 to 50 μg/mL; for Complex B the concentrations ranged from 0.05 to 25 μg/mL. Initial solutions of the complexes were diluted to the desired concentrations in the assay medium and 100 μL of the solution was added to the wells of 96-well plates, then 100 μL of bacterial inoculum was added. Rows 11 and 12 were left for the negative control (medium with inoculum without the metal complex) and sterility control. To assess the contribution of DES to antibacterial activity, its effect on the survival of *S. aureus* was studied separately, without addition of the complexes. Gentamicin (PanEco, Moscow, Russia) was used as a positive control.

The samples were incubated for 24 h. After the incubation period, the MIC endpoints were registered using an iMark microplate absorbance reader (Bio-Rad, Hercules, CA, USA). Absorbance spectra were obtained at λ = 595 nm. The MIC was taken to be the lowest drug concentration that caused complete inhibition of microorganism growth compared to the control.

### 4.4. Statistics

Calculations were performed using the “Descriptive Statistics” data analysis tool in MS Excel. To reflect the twofold serial dilution method, results are presented as the most frequently occurring values ± 95% confidence interval.

### 4.5. EPR Experiments

EPR spectra were recorded with a Bruker (Billerica, MA, USA) Elexsys E-680X radio spectrometer operating at X-band frequency ~9.8 GHz at a temperature of 100 K, modulation amplitude of 5 Gs, and microwave power of 2 mW.

### 4.6. Study of the Transdermal Penetration of the Therapeutic Complex Using a Vertical Franz Diffusion Cell

Franz diffusion cell was used to perform the in vitro permeation studies. The Franz cell was assembled as described in detail in [63] and consisted of donor chamber, acceptor chamber, magnetic stirrer, and a cellulose membrane. Logan’s Permeation-Barrier Membrane (Logan Instruments corp., Somerset, NJ, USA), which is similar in acidity to the human epidermis, was used. A total of 400 mg of the analyzed solution was applied to the donor zone of the membrane. The acceptor chamber was filled with 10 mL of physiological phosphate buffer with pH 7.4. The buffer solution was stirred constantly at 350 rpm and its volume was kept constant; the temperature was maintained at 37 ± 0.5 °C. Sampling was carried out using a syringe with a volume of 1 mL.

### 4.7. ICP-OES

Simultaneously, a Thermo Scientific™ iCAP™ PRO XP ICP-OES (Thermo Fisher Scientific Inc., Waltham, MA, USA) with a vertical torch, a purged echelle polychromator, and Charge Injection Device (CID) array detector, were used to determine content of copper ions in the samples taken from the Franz cell. A standard sample introduction kit suitable for aqueous samples, consisting of a glass cyclonic spray chamber, SeaSpray glass nebulizer, quartz glass duo torch, and other components was used. Instrumental operating conditions are summarized in Table 3.

High-Purity HNO_3_ was used in the digestions. Deionized water with a resistivity of 18.2 MΩ cm at 2 °C was used for all dissolutions and dilutions. Aqueous ICP-OES calibration standards in 5% HNO_3_ were prepared from High-Purity Standards (North Charleston, SC, USA). Argon of 99.996 purity grade was used for ICP-OES measurements.

## Figures and Tables

**Table 1:** MICs values of the tested complexes in different solvents against *S. aureus*.

		*Staphylococcus aureus* MIC ± CI (95%)	
Agent	Solvent	µg/mL	µM
Complex A	DMSO	25 ± 0	53.7 ± 0
	water	25 ± 0	53.7 ± 0
	DES	25 ± 6.42	53.7 ± 13.8
Complex B	DMSO	3.13 ± 0	6.3 ± 0
	water	3.13 ± 0	6.3 ± 0
	DES	3.13 ± 2.11	6.3 ± 4.3
**Control**			
Gentamicin	water	0.5 ± 0.14	1.1 ± 0.3
Complex A with 1% DES	water	25 ± 0	53.7 ± 0
Complex B with 1% DES	water	3.13 ± 0	6.3 ± 0
1% DES	broth medium	Non-toxic	

**Table 2:** Synthesis conditions attempted for production of eutectogels by “in situ” gelation.

	TEOS	TMOS	MTMS
No added catalyst	Immiscible, no stable emulsion.	Immiscible, no stable emulsion.	Initially immiscible, but after 40 h, a sol is formed, with no gelling observed after 1 month.
0.01 M HCl	Immiscible, no stable emulsion.	Initially immiscible, but after 40 h a transparent gel is formed.	Immiscible, no stable emulsion.
CH_3_COOH	Initially immiscible, but after 40 h, a transparent gel is formed.	Initially miscible, but after 40 h, a transparent gel is formed.	Initially miscible, but after 40 h, a sol is formed, with no gelling observed after 1 month.

**Table 3:** ICP-OES operating parameter values.

ICP-OES Operating Parameter	Parameter Value
Forward power, W	1350
Coolant gas flow, L·min^−1^	15
Auxiliary gas flow, L·min^−1^	0.35
Nebulizer gas flow, L·min^−1^	0.65
Pump speed, rpm	60
Radial viewing height, mm	10

## Data Availability

Any raw data can be acquired upon request from the corresponding author.

## Abbreviations

The following abbreviations are used in this manuscript:

DES	Deep eutectic solvent
EPR	Electron paramagnetic resonance
DMSO	Dimethyl sulfoxide
IL	Ionic liquid
API	Active pharmaceutical ingredient
MIC	Minimal inhibitory concentration
TMOS	Tetramethoxysilane
TEOS	Tetraethyl orthosilicate
MTMS	Methyltrimethoxysilane
ICP-OES	Inductively Coupled Plasma Optical Emission Spectrometer
